# Neurofilament Light Chain Concentration in Cerebrospinal Fluid in Children with Acute Nontraumatic Neurological Disorders

**DOI:** 10.3390/children11030360

**Published:** 2024-03-19

**Authors:** Tobias Geis, Svena Gutzeit, Sigrid Disse, Jens Kuhle, Sotiris Fouzas, Sven Wellmann

**Affiliations:** 1 University Children’s Hospital Regensburg (KUNO) at the Hospital St. Hedwig of the Order of St. John, University of Regensburg, 93049 Regensburg, Germany; 2 Department of Neurology, University Hospital, University of Basel, 4001 Basel, Switzerland; 3 Multiple Sclerosis Centre, Research Center for Clinical Neuroimmunology and Neuroscience (RC2NB), Departments of Biomedicine and Clinical Research, University Hospital, University of Basel, 4001 Basel, Switzerland; 4 Department of Pediatrics, University Hospital of Patras, 265 04 Patras, Greece; 5 Department of Neonatology, University Children’s Hospital Regensburg (KUNO) at the Hospital St. Hedwig of the Order of St. John, University of Regensburg, 93049 Regensburg, Germany

**Keywords:** neurofilament light chain NfL, biomarker, meningitis, Bell’s facial palsy, Lyme neuroborreliosis LNB, child

## Abstract

(1) Introduction: This pilot study aimed to analyze neurofilament light chain levels in cerebrospinal fluid (cNfL) in a cohort of children with different acute nontraumatic neurological conditions. (2) Methods: This prospective observational cohort study consisted of 35 children aged 3 months to 17 years and was performed from November 2017 to December 2019. Patients’ clinical data were reviewed, and patients were assigned to the following groups: *n* = 10 (28.6%) meningitis, 5 (14.3%) Bell’s palsy, 7 (20.0%) febrile non-CNS infection, 3 (8.6%) complex febrile seizure, 4 (11.4%) idiopathic intracranial hypertension, and 6 (17.1%) others. cNfL levels were measured using a sensitive single-molecule array assay. (3) Results: The cNfL levels [median (range)] in children with meningitis were 120.5 pg/mL (58.1–205.4), in Bell’s palsy 88.6 pg/mL (48.8–144.5), in febrile non-CNS infection 103.9 pg/mL (60.1–210.8), in complex febrile seizure 56 pg/mL (53.2–58.3), and in idiopathic intracranial hypertension 97.1 pg/mL (60.1–124.6). Within the meningitis group, children with Lyme neuroborreliosis (LNB) had significantly higher cNfL concentrations (median 147.9 pg/mL; range 87.8–205.4 pg/mL) than children with enterovirus meningitis (72.5 pg/mL; 58.1–95.6 pg/mL; *p* = 0.048) and non-significantly higher cNfL levels when compared to Bell’s palsy (88.6 pg/mL; 48.8–144.5 pg/mL; *p* = 0.082). There was no correlation between cNfL levels and age. (4) Conclusions: Although the number of patients in this pilot study cohort is limited, higher cNfL levels in children with LNB compared to those with viral meningitis (significant) and Bell’s palsy (trend) may indicate the potential of cNfL as a biomarker in the differential diagnosis of pediatric meningitis and facial palsy.

## 1. Introduction

Acute pediatric neurological diseases such as meningitis, Bell’s facial palsy, complex febrile seizures, and idiopathic intracranial hypertension (pseudotumor cerebri syndrome) are common diagnoses in children that lead to hospital admission and performance of a lumbar puncture. The CSF of these children is analyzed for inflammatory, immunological, metabolic, and microbiological parameters, and the CSF opening pressure can be measured. Particularly in children with acute facial paralysis, laboratory examination of cerebrospinal fluid (CSF) is mandatory to differentiate between idiopathic Bell’s palsy and Lyme neuroborreliosis (LNB) as the two most common causes in Lyme-endemic areas. LNB is caused by the tick-borne spirochete Borrelia burgdorferi and frequently manifests as isolated acute facial paralysis in pediatric patients requiring antibiotic treatment [1]. On the contrary, idiopathic Bell’s palsy does not require the use of antibiotics, but corticosteroid treatment could be considered. However, the CSF results for these two diseases overlap, and it may take several days to make a definitive diagnosis. Therefore, for differential diagnosis of acute facial paralysis and other pediatric neurological conditions, an early available CSF biomarker would be needed to help shorten the diagnostic process and guide further medical treatment. This could also improve the early and reliable provision of information to patients and their parents, reduce uncertainty and possibly shorten the length of hospitalization. While several biomarkers are well established in pediatric traumatic brain injuries [2], more data are needed on CSF biomarkers in acute nontraumatic neurological disorders in children.

In the past years, neurofilaments (Nf) have gained increasing attention as biomarkers that specifically detect neuroaxonal damage. These proteins are located in neurons of the peripheral and central nervous system and are expressed exclusively by neurons. Depending on their molecular mass, neurofilament light (NfL), medium (NfM), and heavy (NfH) chains can be distinguished [3]. Elevated levels of neurofilaments have been found in various acute and chronic neurological diseases in adults, such as multiple sclerosis, traumatic brain injuries, neurodegenerative dementia, stroke, amyotrophic lateral sclerosis, Parkinson’s disease, and many others [3,4]. Major efforts are being made to establish Nf as a biomarker to monitor disease activity and even response to treatment, as has been shown, for example, in multiple sclerosis, Alzheimer’s disease and autoimmune encephalitis [5,6]. Much less information is available on Nf in pediatric neurological diseases. Recently, however, more pediatric Nf data have emerged, and elevated levels of NfL have been detected in pediatric multiple sclerosis, spinal muscular atrophy, X-linked adrenoleukodystrophy, myelin oligodendrocyte glycoprotein-associated disease (MOGAD), cerebrovascular disease in children with sickle cell anemia, and others [7,8,9,10,11,12]. Moreover, elevated NfL levels in cerebrospinal fluid (cNfL) have been described in a cohort with infectious and inflammatory CNS diseases without providing details on subgroups [13]. NfL concentrations in serum are about 40 times lower than CSF concentrations and are considered a good surrogate measure for cNfL [6]. When interpreting the NfL values obtained in children, it should be noted that there is an age-dependent normal range with highest serum NfL concentrations in infancy, followed by a decline to minimum values at around 10 years of age; thereafter, serum NfL values increase in healthy individuals until old age [14,15].

The aim of this pilot study was to compare cNfL levels in children with various acute nontraumatic neurological diseases to develop a hypothesis for future pediatric studies on cNfL in specific neurological disorders. For the major subgroups of this study population, it was hypothesized that NfL levels would be higher in children with bacterial meningitis than in children with viral meningitis and with Bell’s palsy.

## 2. Material and Methods

### 2.1. Study Cohort

The study cohort recruited for this study comprised pediatric patients who were admitted as inpatients to the University Children’s Hospital Regensburg (KUNO), Klinik St. Hedwig due to an acute neurologic condition. All patients were treated according to the hospital’s standard operating procedures (SOP). Lumbar puncture was usually performed within 24 h of hospital admission. Neurologic diagnoses were made by an experienced pediatric neurologist (TG) based on the patient’s medical history and clinical data. Recruitment of patients was conducted by SG. In most cases, patients and/or their parents were informed about the details of the study on the second or third day of their hospitalization and asked for their consent to participate. Inclusion criteria were age 0 to 17 years, inpatient treatment at the University Children’s Hospital Regensburg (KUNO) Klinik St. Hedwig, (suspected) acute neurologic disease on admission to the hospital leading to performance of a lumbar puncture, and informed consent of legal guardians and of the patients if aged 10 years or older. Exclusion criteria were lack of consent of the legal guardians or language barriers. Children with various acute pediatric neurological diseases at initial diagnosis were included in this study: meningitis and suspected meningitis, Bell’s palsy, complex febrile seizure, idiopathic intracranial hypertension; one patient each with sinus vein thrombosis (SVT), trochlear palsy, pediatric acute neuropsychiatric disorder associated with streptococcus (PANDAS), focal epileptic status, painful neck stiffness, and psychosis. Diagnosis of LNB was made in patients who fulfilled at least two of the following criteria [16]: typical neurological symptoms (in our cohort: facial palsy; meningoradiculitis); elevated CSF leucocytes; intrathecal *B. burgdorferi* IgM, or positive intrathecal *B. burgdorferi* IgG antibody production index. Recruitment of all participants was prospectively conducted between November 2017 and October 2019 after written informed consent had been obtained from the parents as legal guardians. After completion of the regular diagnostic procedures, excess CSF samples were collected and stored at −80 °C. In principle, no additional sampling procedures were carried out for this study alone, and the duration of hospitalization was not extended for the patients and their families as a result of participation in the study.

Approval for the study was granted by the Ethics Committee of the University Regensburg on 24 March 2017 (16-386-101).

### 2.2. NfL Analysis

Concentrations of NfL were measured by single-molecule array (SiMoA) assay performed on the instrument HD-1 Analyzer (Quanterix, Lexington, MA, USA) using the two-step Assay Dilution 2.0 protocol for the NF-light Advantage kit according to the manufacturer’s protocol. Samples were measured in duplicate. Inter-assay variability was evaluated with three native quality control CSF samples in each measurement run. All samples produced signals greater than the analytical sensitivity of the assay. Mean intra-assay variability and inter-assay variability were lower than 10%. NfL analysis in CSF was described in more detail elsewhere [17].

### 2.3. Statistics

Variables are presented as median with range. Comparisons between the groups for age in Table 1 and Table 2 were performed with the Kruskal-Wallis test. Between-group comparisons, were performed with the Mann–Whitney U test, as the data did not follow a normal distribution. Spearman’s rho was used to assess the correlation between cNfL levels and age. In a multivariable regression model including age and CrP, age was not a relevant influencing factor for NfL in the subgroups of our cohort (adjusted R^2^ = 0.029). The model showed no signs of multicollinearity (vif 1.1 for all included covariates).

Analyses were performed with the SPSS software version 27 (IBM, Armonk, NY, USA) and with RStudio 2022.12.0.

## 3. Results

### 3.1. Study Cohort and Groups

CSF samples of 35 participants (18 females, 51%) with an age range from 3 months to 17 years (median 8.0 years) were analyzed. Median body weight was 27.9 kg with a range from 4.9 kg to 115 kg, and median height was 130 cm with a range from 57 cm to 196 cm. Of the 35 participants, 10 (28.6%) were diagnosed with meningitis, 5 (14.3%) with Bell’s palsy, 7 (20.0%) with febrile infection (non-CNS), 4 (11.4%) with idiopathic intracranial hypertension, 3 (8.6%) with complex febrile seizure, and 6 (17.1%) with other diagnoses: one patient each was included with diagnosis of sinus vein thrombosis (SVT), trochlear palsy, pediatric acute neuropsychiatric disorder associated with streptococcus (PANDAS), focal epileptic status, painful neck stiffness, and psychosis.

A microbiological workup of the 10 patients in the meningitis group revealed Lyme neuroborreliosis (LNB) in 6 patients, enterovirus meningitis in 3 children, and pneumococcal meningitis in one patient. Of the six children with LNB, five had isolated facial palsy, and one had polyradiculoneuritis. All participating patients underwent extensive laboratory diagnostics, which showed highly variable C-reactive protein (CRP) values on admission to the hospital, ranging from 0 mg/L to 174 mg/L (median 1.0 mg/L). All patients in the subgroup with febrile non-CNS infection had elevated CRP levels with a median of 28 mg/L (range 6–174 mg/L) compared to the subgroup with complex febrile seizures (median CRP 10 mg/l; range 0–108 mg/L) and the subgroup with meningitis (1.5 mg/L; 0–20 mg/L). Remarkably, the CRP of the patient with pneumococcal meningitis increased from 20 mg/L at the time of hospital admission to a maximum value of 206 mg/L during the further follow-up in the hospital. All patients in the subgroups with Bell’s palsy, idiopathic intracranial hypertension, and other diagnoses (*n* = 15) had a CRP concentration in the normal range. The results of the CSF cell count analysis also varied greatly from individual to individual and differed greatly between the subgroups: For the total cohort, the CSF cell count was a median of 3 cells/µL with a range of 0 to 5600 cells/µL. The CSF cell count was elevated in all patients with meningitis, with a median of 86 cells/µL and a range of 13 to 5600 cells/µL. In all other patients, the CSF cell count was in the normal range between 0 and 4 cells/µL, with the exception of slightly elevated values in two patients with Bell’s palsy (7 and 12 cell/µL, respectively), 18 cells/µL in the psychosis patient, and 11 cells/µL in the PANDAS patients who were treated with intravenous immunoglobulins nine days prior to the lumbar puncture.

The clinical characteristics of the cohort and subgroups are summarized in Table 1 and Table 2.

### 3.2. NfL Levels

**Figure 1:**
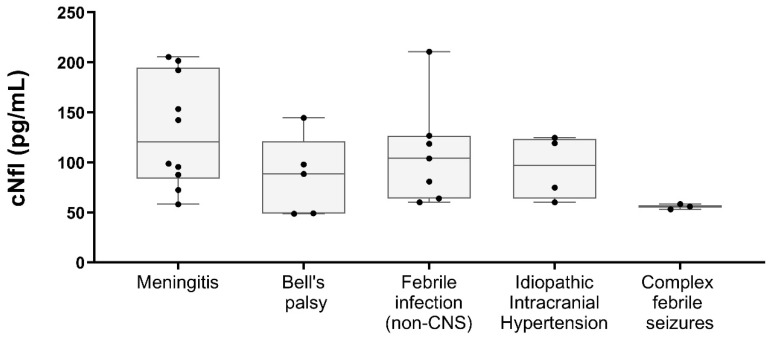
CSF NfL levels in the study groups presented as box and whisker plots.

**Figure 2:**
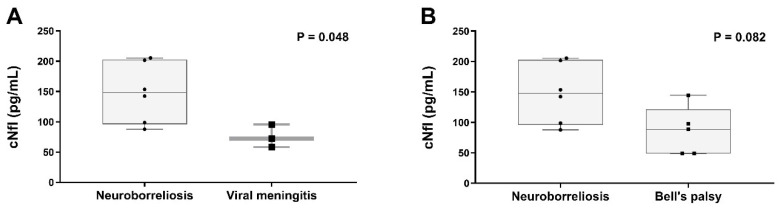
CSF NfL levels in children with neuroborreliosis vs. viral meningitis (**A**) and in children with neuroborreliosis and Bell’s palsy (**B**). Comparisons were performed with Mann–Whitney U test.

**Figure 3:**
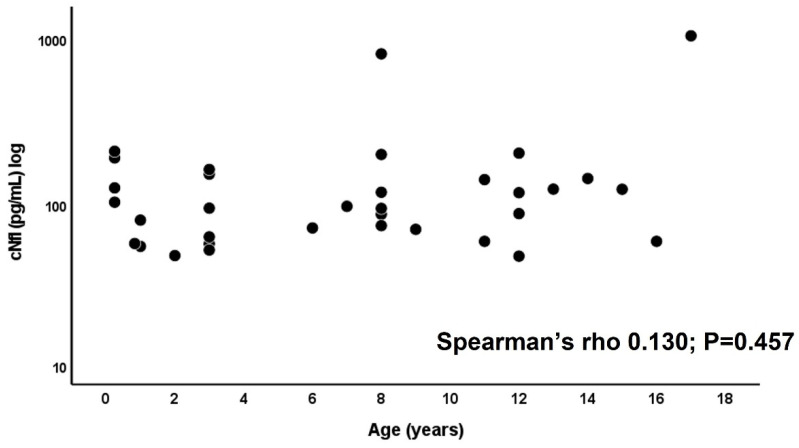
Correlation between cNfL levels and age in our study population.

No significant differences were found between the cNfL values in male (median 87.8 pg/mL, range 53.2–1041.7 pg/mL) and female study participants (median 114.2 pg/mL, range 48.8–811.9 pg/mL; *p* = 0.314). The NfL concentration [median (range)] was 120.5 pg/mL (58.1–205.4 pg/mL) in children with meningitis, 88.6 pg/mL (48.8–144.5 pg/mL) in Bell’s palsy, 103.9 pg/mL (60.1–210.8 pg/mL) in children with febrile infection, 97.1 pg/mL (60.1–124.6 pg/mL) in children with idiopathic intracranial hypertension, and 56 pg/mL (53.2–58.3 pg/mL) in children with complex febrile seizures (Figure 1). A cNfL level of 811.9 pg/mL was measured in the patient with SVT, 163.8 pg/mL in the child with trochlear paresis, 71.1 pg/mL in the PANDAS patient, 95.3 pg/mL in the child with focal epileptic status, 124.4 pg/mL in the patient with neck pain, and 1041.7 pg/mL in the patient with psychosis. There was no significant difference between the cNfL values of children with meningitis and febrile children without meningitis (see Supplementary Figure S1). Of the children with meningitis, those with LNB had significantly higher cNfL concentrations (median 147.9 pg/mL, range 87.8–205.4 pg/mL) than those with enterovirus meningitis (median 72.5 pg/mL, range 58.1–95.6 pg/mL; *p* = 0.048) (Figure 2A). Comparing cNfL levels in children with Lyme neuroborreliosis to those with Bell’s palsy, there was a clear trend toward higher cNfL levels in Lyme neuroborreliosis, but this did not reach a significance level (*p* = 0.082; Figure 2B). There was no correlation between cNfL levels and age in our cohort (Spearman’s rho 0.130; *p* = 0.457) (Figure 3).

We found a moderate, positive correlation between cNfL and CSF protein (Spearman’s rho 0.487; *p* = 0.003) and a weak, positive correlation between cNfL and CSF cell count (Spearman’s rho 0.220; *p* = 0.205). In addition, no or only a weak correlation was found between cNfL and the inflammatory markers CRP and leukocytes in blood.

## 4. Discussion

Laboratory biomarkers are becoming increasingly important for the diagnosis, monitoring and prognosis of numerous diseases. In neurological disorders, NfL is considered a very suitable biomarker as it is highly neuron-specific [3,6]. Further advantages of NfL are a long half-life in body fluids such as CSF and peripheral blood of about 3 to 4 weeks and pre-analytical long-term stability in stored CSF, serum and plasma samples of 7 days at room temperature, more than 10 years at −80 °C as well as in dried blood spot samples for potentially long time periods [18,19,20,21]. The fourth-generation single-molecule array (SiMoA) technology for NfL measurements is highly sensitive and allows exact quantification even of low NfL sample concentrations with a lower limit of 0.8 ng/L [3,22]. Even more, a fully automated assay with a test run time of 45 min has been developed, which could represent an important step toward clinical application of NfL analysis [22].

NfL studies in the pediatric population often focus on multiple sclerosis [6,23]. Therefore, the aim of this pilot study was to examine a cohort of children with different acute nontraumatic neurological conditions to hypothesize future studies of NfL as a biomarker in specific neurological diseases in the pediatric population. Despite the limited number of patients, a main result in our cohort was that cNfL was significantly higher in children with LNB than in children with viral meningitis caused by enteroviruses. The clinical picture with headache, fatigue, and neck stiffness as the main complaints may be very similar in LNB and viral meningitis [1]. Common diagnostic parameters, such as CSF pleocytosis and protein, determined in the initial CSF analysis, do not reliably distinguish between these two etiologies. Therefore, an early available biomarker would be needed to guide the therapy approach when deciding on antibiotic treatment. In line with our study, elevated cNfL concentrations have been described in adults with LNB, and an association between cNfL levels and disease manifestation severity was found, with cNfL levels significantly higher in patients with CNS involvement than in patients in whom only cranial nerves were affected, such as in facial palsy [24,25]. Most recently, Mens et al. speculated whether NfL might be a useful biomarker of response to LNB treatment, as their study in adults revealed a highly significant decrease of plasma NfL at 3 and 6 months after antibiotic treatment of LNB [26]. Similarly, elevated NfL concentrations in CSF and serum in adults with severe community-acquired bacterial meningitis were reported and associated with an unfavorable outcome [27,28]. In an animal model of experimental pneumococcal meningitis, NfL levels in CSF and serum increased 26- and 3.5-fold, respectively, within 18 h post-infection. Both CSF and serum values were strongly correlated, which may open the possibility of follow-up with repeated NfL analysis from blood samples [29]. In children, the CSF concentrations of the neurofilament heavy chain (NfH) were increased in bacterial meningitis compared with controls, with higher NfH long-term levels in children with neurological sequelae [30].

Moreover, NfL might be useful as a biomarker in children diagnosed with acute facial nerve palsy. LNB in children often manifests as acute facial nerve palsy and should be differentiated from idiopathic Bell’s palsy. In a Lyme-endemic region, as many as 43% of pediatric facial nerve palsies were caused by LNB compared to 57% of idiopathic Bell’s palsy [31]. In a study of predominantly adult patients with facial nerve palsy, cNfL levels were significantly higher in the LNB group than in the Bell’s palsy group [25]. It could be hypothesized that a longer pre-symptomatic disease duration from tick bite to the clinical manifestation of facial palsy could be the cause for this. In our pilot study, there was a clear trend toward higher cNfL values in LNB, but without reaching a significance level. However, due to the limited number of participants, a significant effect may have been missed. Furthermore, this result needs to be confirmed in a pediatric study cohort with sufficient power for a proper statistical analysis.

In our small cohort, there was no correlation between cNfL levels and age. Whereas in healthy adults, CSF NfL levels increase by approximately 3.3% per year from young adulthood to old age [5], no normative values are available for cNfL in the pediatric population. However, serum NfL levels in children and adolescents show a biphasic age dependency, with the highest levels in infancy and late adolescence and the lowest levels at about 10 years of age [14]. It can be assumed that the cNfL levels in healthy children and adolescents run parallel to the serum NfL curve. Further data on cNfL concentrations in different pediatric age groups are desirable to establish a reference database similar to the recently established serum NfL reference database [14]. In our study, as described above, the cNfL values were significantly higher in LNB patients than in enterovirus patients. Since the median age was lower in the enterovirus subgroup than in the LNB subgroup (Table 2), the difference in NfL values between these two subgroups could be more distinct after adjustment for age.

In the four patients of our cohort diagnosed with idiopathic intracranial hypertension, CSF opening pressures ranging from 20 to 47 cm H_2_O were measured and did not correlate with cNfL concentrations. Two recent studies addressed cNfL as a biomarker in adults with idiopathic intracranial hypertension and revealed a strong positive correlation with the degree of papilledema and CSF opening pressure [32,33]. Thus, more data on the effects of intracranial pressure on cNfL levels in children is needed to clarify the role of NfL in pediatric idiopathic intracranial hypertension.

By far, the two highest cNfL values in our cohort were measured in one patient with sinus vein thrombosis (811.9 pg/mL) and a second patient with psychosis (1041.7 pg/mL). The latter patient had mild cognitive impairment and T2-hyperintense lesions on cerebral magnetic resonance imaging (MRI). The MRI changes were reminiscent of multiple sclerosis but did not meet the criteria for an MS diagnosis. An underlying chronic neurologic disorder was suspected, but advanced diagnostic testing failed to establish a definitive diagnosis. The girl with sinus vein thrombosis initially presented with headache, abducens nerve paresis and papilledema. No visual field defects and no structural lesions on cerebral MRI were detected.

Strengths of our study include the age range of the participants from infancy to adolescence and the diversity of different pediatric neurologic conditions, providing for the first time new data on cNfL levels in different pediatric neurologic disorders such as meningitis caused by different pathogens, Bell’s palsy, and idiopathic intracranial hypertension.

However, there are considerable limitations. First, the number of participants in this study was limited and the cohort is heterogeneous, allowing only a description of cNfL levels in subgroups with specific diseases but hardly any advanced statistical analysis. Second, NfL was measured only in CSF samples and no serum samples were obtained and analyzed. Third, the cross-sectional study design does not allow evaluation of the temporal NfL profile for different disorders. A future study with suitable design and ethical approval.could focus on NfL measurement in serum and cerebrospinal fluid at different time points in pediatric patients with neurological diseases.

## 5. Conclusions

NfL concentrations in the CSF of children and adolescents with various acute neurological diseases were highly variable. However, although the number of patients in this pilot study cohort was limited, our results suggest that NfL is a promising biomarker in the field of acute nontraumatic neurological disorders such as facial palsy and meningitis in the pediatric population. Further studies should investigate whether NfL could be a valuable biomarker for differential diagnosis in children with meningitis caused by different pathogens, LNB and Bell’s palsy. In addition, the correlation of NfL with disease severity in pediatric meningitis and the potential of NfL as a biomarker for monitoring response to treatment could be investigated in future studies.

## Figures and Tables

**Table 1:** Characteristics of the study population by diagnosis. There were no significant age differences between the main diagnostic subgroups (*p* = 0.072).

		Diagnostic Subgroups					
		Meningitis	Bell’s Palsy	Febrile Infection (Non-CNS)	Idiopathic Intracranial Hypertension	Complex Febrile Seizures	Others
*n*	35	10	5	7	4	3	6
Male sex, *n* (%)	17 (48.6)	5 (50)	0 (0)	3 (42.9)	2 (50.0)	3 (100)	4 (66.7)
Age, years	8.0 (0.3–17)	6.5 (0.3–12.0)	12.0 (2–14)	1.0 (0.3–16.0)	9.5 (8–13)	1.0 (0.83–3.0)	8.5 (3.0–17.0)
BMI	17.1 (13.4–29.9)	16.5 (13.6–18.7)	21.3 (14.5–28.1)	17.4 (15.2–22.7)	19.8 (17.1–21.9)	13.4 (13.4) *	17.8 (15.9–29.9)
CRP, mg/L	1.0 (0–174)	1.5 (0–20)	0 (0–5)	28 (6–174)	1.5 (0–2)	10 (0–108)	0.5 (0–1.0)
CSF cell count, /µL	3 (0–5600)	86 (13–5600)	3 (1–12)	2 (1–4)	1 (0–1)	2 (1–3)	2.5 (1–18)
CSF protein, mg/dL	22.8 (1.31–560.7)	33.4 (13.1–560.7)	25.0 (13.0–37.8)	21.4 (17.7–32.8)	24.7 (19.5–30.0)	18.8 (13.6–25.5)	18.5 (12.0–187.3)
CSF glucose, mg/dL	56.0 (4.0–99.0)	54.5 (4.0–64.0)	55.0 (50.0–63.0)	64.0 (52.0–77.0)	54.5 (50.0–55.0)	71.0 (59.0–99.0)	54.5 (49.0–57.0)

Data presented as median (range). * values for range and median are identical due to two missing values.

**Table 2:** Characteristics of the meningitis subgroups.

	Lyme Neuroborreliosis	Enterovirus Meningitis	Pneumococcal Meningitis
*N*	6	3	1
Male sex, *n* (%)	3 (43)	1 (33)	1 (100)
Age, years	8 (3–12)	3.0 (3–6)	0.25
BMI	16.7 (14.2–18.7)	15.2 (13.6–16.8) *	13.8
CRP, mg/L	1 (0–8)	3 (2–5)	20
CSF cell count, /µL	69 (13–140)	280 (85–719)	5600
CSF protein, mg/dL	26.8 (13.1–94.7)	36.0 (22.8–41.8)	560.7
CSF glucose, mg/dL	56 (53–64)	51 (49–57)	4

Data presented as median (range). * Only two values are available.

## Data Availability

The data presented in this study are available on request from the corresponding author. The data are not publicly available due to organizational reasons.

## Supplementary Materials

The following supporting information can be downloaded at: https://www.mdpi.com/article/10.3390/children11030360/s1, Figure S1: Comparison of cNfL in febrile children with and without meningitis. Table S1. Detailed clinical data of all study participants.

## Abbreviations

NfL	Neurofilament light chain
cNfL	Nfl in cerebrospinal fluid
CSF	Cerebrospinal fluid
LNB	Lyme neuroborreliosis
MRI	Magnetic resonance imaging
MS	Multiple sclerosis
