# Supplementary material for: Neurofilament Light Chain Concentration in Cerebrospinal Fluid in Children with Acute Nontraumatic Neurological Disorders
Source: Children (Basel). 2024 Mar 19;11(3):360. doi: 10.3390/children11030360 (PMC10969464; doi:10.3390/children11030360)
Supplement: Supplementary file 1 [file children-11-00360-s001.zip › children-2838159-supplementary.pdf]

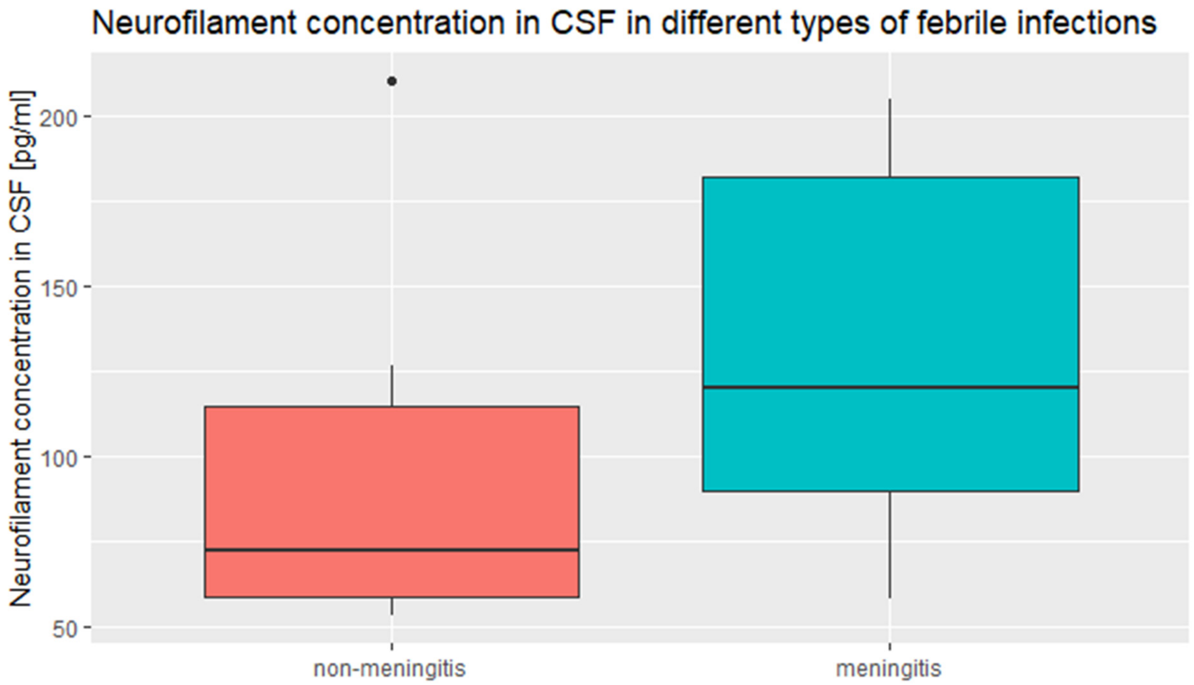

Figure S1:

Comparison of cNfL levels of the meningitis patients (n = 10) with the group of all febrile non-meningitis children (n = 10; complex febrile seizure + febrile infection). There was no significant difference between cNfL levels in children with meningitis (median 120.5 pg/mL; range 58.1-205.4 pg/mL) and febrile non-meningitis children (median 72.5 pg/ml; range 53.3-210.8; p = 0.14).
